# Genomic Surveillance of Carbapenem-Resistant Klebsiella pneumoniae from a Major Public Health Hospital in Singapore

**DOI:** 10.1128/spectrum.00957-22

**Published:** 2022-09-06

**Authors:** Jocelyn Qi-Min Teo, Cheng Yee Tang, Si Hui Tan, Hong Yi Chang, Sze Min Ong, Shannon Jing-Yi Lee, Tse-Hsien Koh, James Heng-Chiak Sim, Andrea Lay-Hoon Kwa, Rick Twee-Hee Ong

**Affiliations:** a Department of Pharmacy, Singapore General Hospital, Singapore, Singapore; b Saw Swee Hock School of Public Health, National University of Singapore and National University Health System, Singapore, Singapore; c Department of Pharmacy, National University of Singapore, Singapore, Singapore; d Department of Microbiology, Singapore General Hospital, Singapore, Singapore; e Singhealth Duke-NUS Medicine Academic Clinical Programme, Singapore, Singapore; f Emerging Infectious Diseases, Duke-National University of Singapore Medical School, Singapore, Singapore; University Paris-Saclay, AP-HP Hôpital Antoine Béclère, Service de Microbiologie, Institute for Integrative Biology of the Cell (I2BC), CEA, CNRS

**Keywords:** carbapenem-resistant, *Klebsiella pneumoniae*, whole-genome sequencing, drug resistance mechanisms, plasmid-mediated resistance, virulence factors

## Abstract

Carbapenem-resistant Klebsiella pneumoniae (CRKP) is a global public health threat. In this study, we employed whole-genome sequencing (WGS) to determine the genomic epidemiology of a longitudinal collection of clinical CRKP isolates recovered from a large public acute care hospital in Singapore. Phylogenetic analyses, a characterization of resistance and virulence determinants, and plasmid profiling were performed for 575 unique CRKP isolates collected between 2009 and 2020. The phylogenetic analyses identified the presence of global high-risk clones among the CRKP population (clonal group [CG] 14/15, CG17/20, CG147, CG258, and sequence type [ST] 231), and these clones constituted 50% of the isolates. Carbapenemase production was common (*n* = 497, 86.4%), and KPC was the predominant carbapenemase (*n* = 235, 40.9%), followed by OXA-48-like (*n* = 128, 22.3%) and NDM (*n* = 93, 16.2%). Hypervirulence was detected in 59 (10.3%) isolates and was most common in the ST231 carbapenemase-producing isolates (21/59, 35.6%). Carbapenemase genes were associated with diverse plasmid replicons; however, there was an association of *bla*_OXA-181/232_ with ColKP3 plasmids. This study presents the complex and diverse epidemiology of the CRKP strains circulating in Singapore. Our study highlights the utility of WGS-based genomic surveillance in tracking the population dynamics of CRKP.

**IMPORTANCE** In this study, we characterized carbapenem-resistant Klebsiella pneumoniae clinical isolates collected over a 12-year period in the largest public acute-care hospital in Singapore using whole-genome sequencing. The results of this study demonstrate significant genomic diversity with the presence of well-known epidemic, multidrug-resistant clones amid a diverse pool of nonepidemic lineages. Genomic surveillance involving comprehensive resistance, virulence, and plasmid gene content profiling provided critical information for antimicrobial resistance monitoring and highlighted future surveillance priorities, such as the emergence of ST231 K. pneumoniae strains bearing multidrug resistance, virulence elements, and the potential plasmid-mediated transmission of the *bla*_OXA-48-like_ gene. The findings here also reinforce the necessity of unique infection control and prevention strategies that take the genomic diversity of local circulating strains into consideration.

## INTRODUCTION

Klebsiella pneumoniae, a key member of the Enterobacterales family, is a medically important pathogen that is implicated in several nosocomial infections, including urinary tract infections and pneumonia. It is also a commonly encountered organism in intensive care units (1). This pathogen is a major public health threat, owing to its ability to acquire multidrug resistance, including carbapenem resistance. The emergence of carbapenem-resistant K. pneumoniae (CRKP) has been singled out as a leading priority by the World Health Organization and the United States Centers for Disease Control and Prevention (2, 3).

Singapore’s strategic geographical location has positioned it as an international hub for travel, trade, and medical tourism. However, overseas travel has also provided the opportunity for the importation of diverse, novel, multidrug resistant (MDR) bacteria into the country (4, 5). Coupled with the endemicity of extended-spectrum *β*-lactamases (ESBLs) and the corresponding high carbapenem usage, it is not surprising that a dramatic increase in the incidence of carbapenem-resistant Enterobacterales (including K. pneumoniae) was observed (6). Among the Enterobacterales, K. pneumoniae presented with the highest carbapenem resistance rates; the national prevalence of CRKP in 2017 was 7.0 per 100 000 patient days, more than twice that of carbapenem-resistant E. coli (7).

Antimicrobial resistance (AMR) has emerged significantly in certain clones and clonal groups (CGs). The global dissemination of carbapenem resistance is associated with a limited number of successful “high-risk” clones. These problematic clones are defined as global high-risk clones based primarily on the following characteristics: (i) isolation from various international geographical locations; (ii) multidrug resistance; (iii) enhanced fitness, virulence, and pathogenicity; (iv) extended host colonization and persistence; and (v) extensive transmission among hosts (8, 9). The most recognized international clones belong to CG258, which includes sequence type (ST) 258, ST11, and ST512. These strains are commonly implicated in the dissemination of KPC-2/KPC-3 carbapenemases, especially in the United States and Israel. Other common high-risk clones include those belonging to CG14/15, CG17/20, CG43, CG147, and ST101 (10, 11).

Of even greater concern is the genotypic convergence of carbapenem resistance and virulence. Multi-drug resistance and hypervirulence were often deemed to have followed distinct evolutionary directions, with each phenotype occupying its own clonal lineage and possessing a largely nonoverlapping genomic signature (12). AMR often occurs in less virulent classical pathotypes that are encountered more frequently in healthcare settings (13). On the other hand, well-known hypervirulent K. pneumoniae (hvKP) clones (e.g., ST23, ST25, ST65, and ST86) are mostly implicated in severe community-acquired infections and are often highly susceptible to antibiotics (14). However, instances of the acquisition of virulence genes by CRKP and the acquisition of resistance genes by hvKP are increasingly being reported, posing a significant public health challenge (15, 16).

Limited evidence has pointed toward the high dynamicity and diversity of carbapenem-resistant Enterobacterales locally (4, 6, 17). Such diversity can introduce complexities in the management of these resistant organisms. Contemporary surveillance data would thus be beneficial in deciphering AMR trends, informing public health policies and interventions, guiding treatment selection, and assessing the impact of interventions (18). Therefore, we aimed to use whole-genome sequencing (WGS) to understand the population structure and the resistance and virulence determinants of CRKP, the predominant carbapenem-resistant Enterobacterales in Singapore, from samples collected between 2009 and 2020 from a local public health hospital.

## RESULTS

### CRKP isolates and antibiotic susceptibilities.

**FIG 1:**
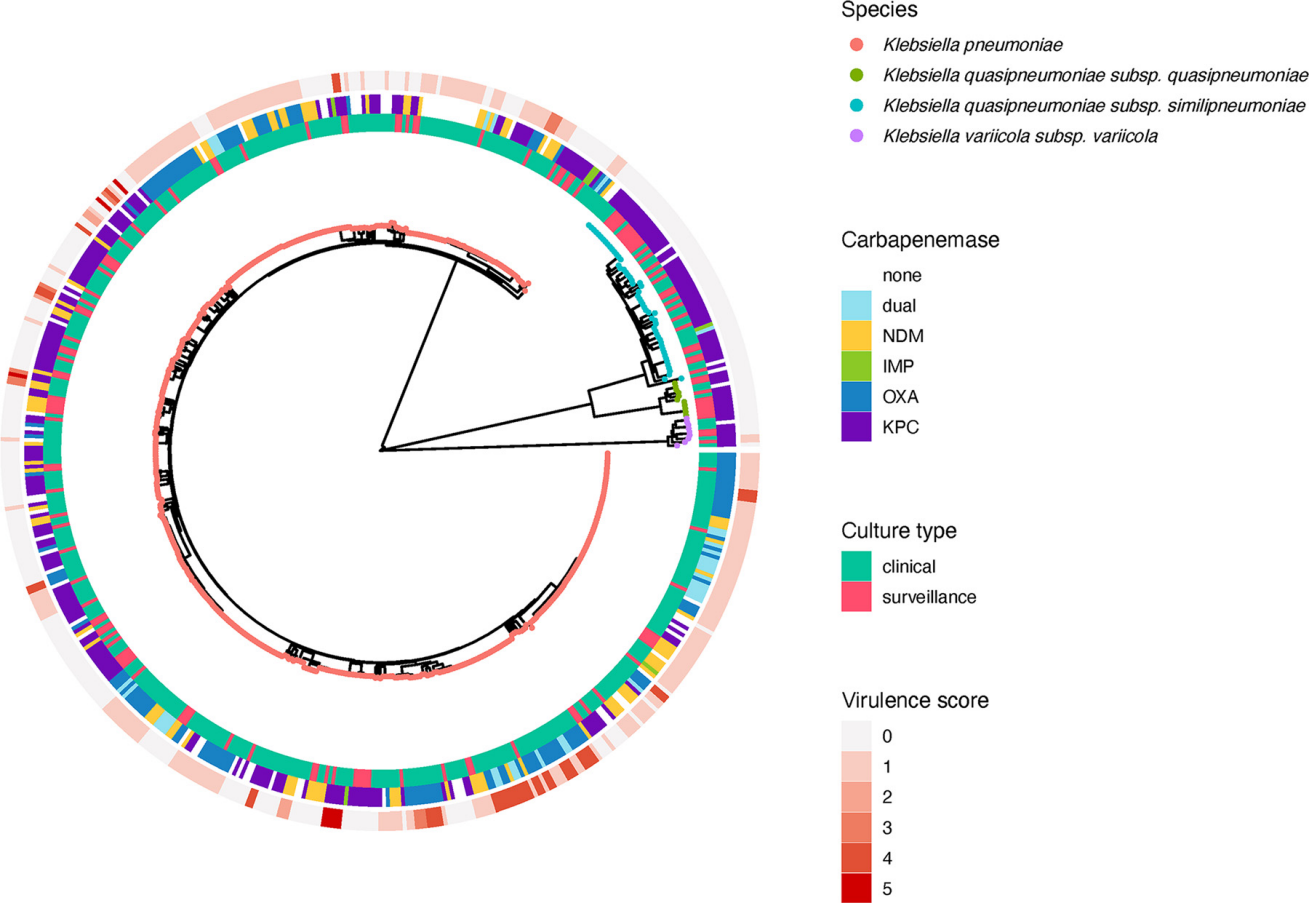
Core SNP phylogenetic tree of 575 carbapenem-resistant K. pneumoniae genomes. Tree tips are color-labeled by K. pneumoniae subspecies. The culture type (innermost ring), carbapenemase type (middle ring), and Kleborate virulence score (outermost ring) are annotated according to the legend.

*In silico* species identification showed that K. pneumoniae *sensu stricto* (*n* = 500, 87.0%) accounted for the majority of the isolates, followed by *K. quasipneumoniae* subsp. *similipneumoniae* (*n* = 55, 9.6%), *K. quasipneumoniae* subsp*. quasipneumoniae* (*n* = 11, 1.9%), and *K. variicola* subsp. *variicola* (*n* = 9, 1.6%). Similar to previous population structure studies of K. pneumoniae, we observed three distinct phylogroups corresponding to these three species groups: KpI (K. pneumoniae *sensu stricto*), KpII (*K. quasipneumoniae*), and KpIII (*K. variicola*) (indicated by the different colored nodes in Fig. 1) (10).

CRKP was resistant to multiple antibiotic classes, whereas most isolates were resistant to levofloxacin (66.4%) in addition to the various β-lactams. Lower resistance rates were observed for amikacin (25.2%), polymyxin B (11.1%), and tigecycline (4.2%) (Table 1). The antibiotic susceptibilities of the KpI and non-KpI isolates were comparable, except for cefepime, levofloxacin, and amikacin; KpI isolates had higher cefepime susceptibility rates (5.8% versus 0%, *P* = 0.032) but lower amikacin (69.0% versus 97.3%, *P* < 0.001) and levofloxacin susceptibility rates (17.0% versus 48.0%, *P* < 0.001).

**TABLE 1:** Antibiotic susceptibility patterns of 575 carbapenem-resistant K. pneumoniae^*a*^

Antibiotic	% S	% I/SDD	% R	MIC data (mg/L)		
				MIC_50_	MIC_90_	Range
Ertapenem	0.0	1.6	98.4	≥32	≥32	≤1 to ≥32
Doripenem	7.0	5.9	87.1	16	≥32	≤0.5 to ≥32
Imipenem	5.6	4.0	90.4	16	≥32	≤0.5 to ≥32
Meropenem	6.6	5.6	87.8	≥32	≥32	≤0.5 to ≥32
Aztreonam	4.9	1.4	93.7	≥64	≥64	≤2 to ≥64
Cefepime	5.0	11.0	84.0	≥64	≥64	≤1 to ≥64
Piperacillin-tazobactam	0.4	3.1	96.5	≥128/4	≥128/4	8/4 to ≥128/4
Levofloxacin	21.1	12.5	66.4	16	≥64	≤0.25 to ≥64
Amikacin	72.7	2.1	25.2	8	≥128	≤4 to ≥128
Polymyxin B	-	88.9	11.1	0.5	4	≤0.25 to ≥16
Tigecycline	88.3	7.5	4.2	1	4	≤0.25 to ≥16

a The categorical susceptibilities were determined using CLSI breakpoints, except for tigecycline (FDA). S, susceptible; I, intermediate; SDD, susceptible dose-dependent; R, resistant; MIC, minimum inhibitory concentration.

### Clonal diversity and phylogenetic analyses.

There was significant diversity observed among the K. pneumoniae species. 151 distinct STs were identified in this study, including 22 novel STs which have been submitted to the Institute Pasteur K. pneumoniae MLST database. The prevalence for each ST ranged from a single isolate to 54 isolates (ST14, 9.4%). Other predominant STs include: ST147 (*n* = 41, 7.1%), ST16 (*n* = 39, 6.8%), and ST231 (*n* = 33, 5.7%).

Based on the goeBURST analyses using the criteria where all members assigned to the same group are single locus variants of each other, 52 different STs were grouped into 20 CGs. The remaining 99 STs were singletons (single STs that do not correspond to any CG). The largest CGs were CG14/15, which consisted of ST14, ST15, ST709, and ST2096 (*n* = 79, 13.7%), and CG17/20, which consisted of ST16, ST17, ST20, and ST3247 (*n* = 78, 13.6%). Other noteworthy groups include CG147 (ST147, ST273, ST392, ST885, ST4843, ST5612, and ST5613) (*n* = 56, 9.7%), CG258 (ST11, ST258, ST340, and ST437) (*n* = 39, 6.8%), and ST231 (*n* = 33, 5.7%). Together, these CGs represented only 49.6% of the study population. The high diversity is emphasized by the large numbers of STs which were represented by only one isolate (*n* = 93/151, 61.6%). The high-risk MDR global clones ST15, ST20, ST37, ST101, ST147, ST258, and ST307, as well as the known hvKP clones ST23, ST25, ST65, and ST86 were detected in our population (19).

While CG17/20 was equally prevalent in both the clinical and the surveillance isolates, the other dominant CG/STs in clinical isolates were less frequently observed in the surveillance isolates (CG14/15: 6.8% versus 15.8%, *P* = 0.009; CG258: 1.5% versus 8.4%, *P* = 0.005; CG147: 4.5% versus 11.3%, *P* = 0.022; ST231: 2.3% versus 6.8%, *P* = 0.055). Instead, ST477 was observed frequently (8.3% versus 0.9%, *P* < 0.001) in the surveillance isolates, in view of the overrepresentation of *K. quasipneumoniae* in this cohort.

### Carbapenemase production.

Overall, carbapenemase production was detected in 497 isolates (86.4%). KPC was the predominant carbapenemase (*n* = 235, 40.9%), followed by OXA-48-like (*n* = 128, 22.3%) and NDM enzymes (*n* = 93, 16.2%). A small proportion (*n* = 35, 6.1%) harbored two carbapenemases concurrently. Of these, most were carrying OXA-48-like enzymes in combination with NDM.

**FIG 2:**
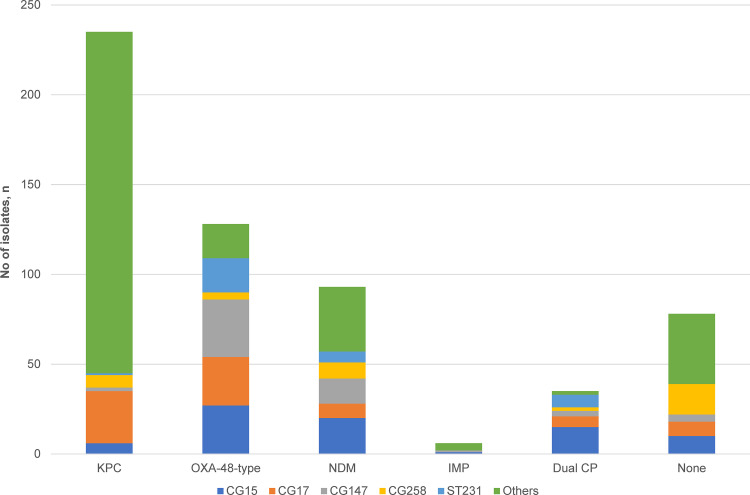
Distribution of carbapenemases among the major clonal groups in carbapenem-resistant K. pneumoniae. The carriage of OXA-48-type enzymes was associated with CG14/15 (*P ≤ *0.001), CG17/20 (*P* = 0.002), CG147 (*P ≤ *0.001), and ST231 (*P ≤ *0.001). The carriage of NDM enzymes was associated with CG14/15 (*P ≤ *0.001) and ST231 (*P* = 0.015). KPC carriage was not associated with any of the clonal groups. The *P* values indicated here are determined by chi-square tests for two-group comparisons of each CG/ST versus all other CG/STs. Abbreviations: CG, clonal group; CP, carbapenemase; ST, sequence type.

Carbapenemases were carried by diverse clones. KPC carbapenemases were disseminated by an array of clones (*n* = 103). Among the KPC-producers (all harbored KPC-2), a significant proportion was borne by non-KpI isolates (67/235, 28.5%; *P* < 0.001) (Fig. 1). The non-KpI species, *K. quasipneumoniae* and *K. variicola*, were predominantly KPC-producers (67/75, 89.3%). The NDM-producing (NDM-1, NDM-4, NDM-5, NDM-7, and NDM-9) isolates were distributed across 31 STs and displayed less diversity than the KPC-producers (distributed across 103 STs), although the predominant STs (e.g., ST14, ST15, and ST147) accounted for 61% of the NDM-carrying isolates (Fig. 2). The diversity of strains carrying OXA-48-like carbapenemases and those harboring two carbapenemases concurrently were even lower (distributed across 23 STs); isolates belonging to CG14/15, CG17/20, CG147, and ST231 accounted for 84% of the OXA-48-like population (Fig. 2). Among these OXA-48-like-carriers, the frequency of *bla*_OXA-232_ was the highest (67/161, 41.6%), followed by *bla*_OXA-181_ (51/161, 31.7%) and *bla*_OXA-48_ (43/161. 26.7%).

In addition, the distribution of carbapenemases differed between the clinical and the surveillance isolates. While the carbapenemase types were more evenly distributed among the 443 clinical isolates (OXA-48-like: 33.9%, KPC: 33.6%, NDM: 21.0%), there was an overrepresentation of KPCs (88, 66.7%) in the 132 surveillance isolates. The NDM and OXA-48 prevalence in the surveillance isolates were 26.5% and 8.3%, respectively.

### Other AMR determinants.

Acquired resistance genes to the various classes were identified in the majority of the isolates. The median number of resistance genes identified was 12 (range: 1 to 27), encompassing a median of nine antibiotic classes (range: 1 to 12).

ESBL-encoding genes were detected in 392 (68.1%) isolates, and *bla*_CTX-M-15_ was the most prevalent (328/392, 83.7%). We observed multiple ESBL genes in 16 isolates. Plasmidic *ampC* genes (including *bla*_DHA_ and *bla*_CMY_) were far less common, occurring only in 66 (11.5%) isolates. Aside from carbapenemases, carbapenem resistance is also mediated by the combination of ESBLs and porin loss in 231 (40.2%) of the isolates, consisting of 176/497 (35.4%) of the carbapenemase-producing isolates and 55/78 (70.5%) of the non-carbapenemase producing isolates.

The prevalence of acquired genes mediating resistance to aminoglycosides (*n* = 467, 81.2%) and fluoroquinolones (*n* = 374, 65.0%) were equally high. Of note, the prevalence of 16S rRNA methyltransferases (16S RMTases), enzymes capable of mediating broad-spectrum aminoglycoside resistance, was relatively high (143/575, 24.9%). The distribution of the 16S RMTase genes were as follows: *armA* (72/143, 50.3%), *rmtF* (54/143, 37.8%), *rmtB* (24/143, 16.8%), and *rmtC* (5/143, 3.5%). 16S RMTases were primarily associated with isolates carrying OXA-48-like (71/143, 49.7%), NDM (32/143, 22.4%), or both carbapenemases (33/143, 23.1%), and they were overrepresented in ST43 (6/7, 85.7%; *P* < 0.001), ST231 (27/33, 81.8%; *P* < 0.001), and ST14 (38/54, 70.4%; *P* < 0.001). The prevalence of 16S RMTases was lower in the ST147 isolates (16/41, 39.0%), although this sequence type has been associated with the cocarriage of NDM and 16S RMTases in both Spain and the United States (20, 21).

Acquired AMR determinants mediating resistance to last-line agents polymyxin, tigecycline, and fosfomycin appeared to be rare. Polymyxin resistance-mediating *mcr* genes (*mcr*1.1, *mcr*8.2, *mcr*9.1, *mcr*9.2, and *mcr*10.1) were detected in 11 (1.9%) isolates. We noted that phenotypic resistance (polymyxin B minimum inhibitory concentration [MIC] > 2 mg/L) was only detected in the three *mcr*1.1 isolates, whereas the isolates with the other *mcr* variants had low MICs (0.5 to 2 mg/L). Tigecycline resistance-mediating *tet*(X4) genes were detected in only two (0.4%) isolates. This is much lower than the fecal carriage rate (10%) reported in another local study involving healthy volunteers (22). All isolates harbored chromosomal *fos*A, but plasmid-mediated *fos* genes (*fos*A3 and *fos*A4) were detected in only 12 (2.1%) isolates. High fosfomycin MICs were observed for all 12 isolates (11 isolates, MIC ≥ 2048mg/L; 1 isolate, MIC = 16mg/L).

### Virulence-associated genetic determinants.

We detected 83 different known K loci encoding the core capsule biosynthesis machinery, with KL51 (*n* = 70, 12.2%), KL2 (*n* = 46, 8.0%), KL64 (*n* = 45, 7.8%), KL15 (*n* = 30, 5.2%), and KL10 (*n* = 29, 5.0%) representing the most common loci. K1, the capsular serotype associated with K. pneumoniae abscesses, was rare and was found exclusively in the five ST23 hvKP isolates observed in this study. Of these, three of the isolates have been extensively profiled previously (23). We did not observe any strong association of K serotypes with phylogenetic lineages, which paralleled previous observations, in which K serotypes may be associated with several clones and vice versa (24). There were far fewer distinct O loci observed (*n* = 11), with O1 (*n* = 213, 37.0%), O2 (106, 18.4%), and O3b (84, 14.6%) representing the most common loci in concordance with previous reports (25).

The prevalence of the other virulence factors is displayed in Table 2. The gene encoding yersiniabactin (*ybt*), which was associated with 9 different chromosomally integrated mobile elements (ICEKp) and two plasmids, was detected in 202 (41.6%) isolates. ICEKp3, ICEKp4, and ICEKp5 were the most common among these mobile elements. The gene encoding aerobactin (*iuc*), which was used to define hypervirulence in this study, was detected in 59 (10.3%) isolates. All 59 isolates harbored carbapenemase-encoding genes, denoting the convergence of hypervirulence and multidrug resistance. Aside from the known hvKP lineages (ST23, ST65, and ST86), the presence of *iuc* was observed primarily in the ST231-K51/O1 strains (21/59, 35.6%), all of which harbored *bla*_OXA-181/232_ and/or *bla*_NDM_. The remaining virulence genes (*iro*, *clb*, *rmpA*, and *rmpA2*), were far less prevalent, occurring in only 2.4 to 4.5% of the isolates.

**TABLE 2:** Virulence characteristics of 575 carbapenem-resistant K. pneumoniae isolates

Isolate type	No. of isolates	% prevalence					Kleborate virulence score		
		ybt	clb	iuc	iro	rmp/rmp2	Mean	Median	Range
All isolates	575	46.4	2.4	10.3	3.7	4.5	0.8	0	0 to 5
KPC	235	18.7	4.7	6.8	6.8	6.4	0.4	0	0 to 5
OXA	128	88.3	0	23.4	0	3.9	1.6	1	0 to 4
NDM	93	58.1	3.2	6.5	4.3	5.4	0.8	1	0 to 4
IMP	6	16.7	0	0	0	0	0.2	0	0 to 1
Dual carbapenemase	35	74.3	0	14.3	0	0	1.2	1	0 to 4
No carbapenemase	78	37.2	0	2.6	1.3	1.3	0.5	0	0 to 4
Clinical isolates	443	54.0	2.7	12.4	3.8	5.0	0.9	1	0 to 5
Surveillance isolates	132	21.2	1.5	3.0	3.0	3.0	0.3	0	0 to 5

Virulence factors were absent in all non-KpI isolates, except for two *ybt*+ *K. variicola* isolates. This was also reflected by the generally low virulence scores in this group (Fig. 1), which suggested a limited virulence potential in these species and was in concordance with earlier studies that reported low virulence frequencies in these species (26, 27). Similarly, the surveillance isolates generally possessed fewer virulence factors and corresponding lower virulence scores (Table 2).

### Plasmid analyses.

**FIG 3:**
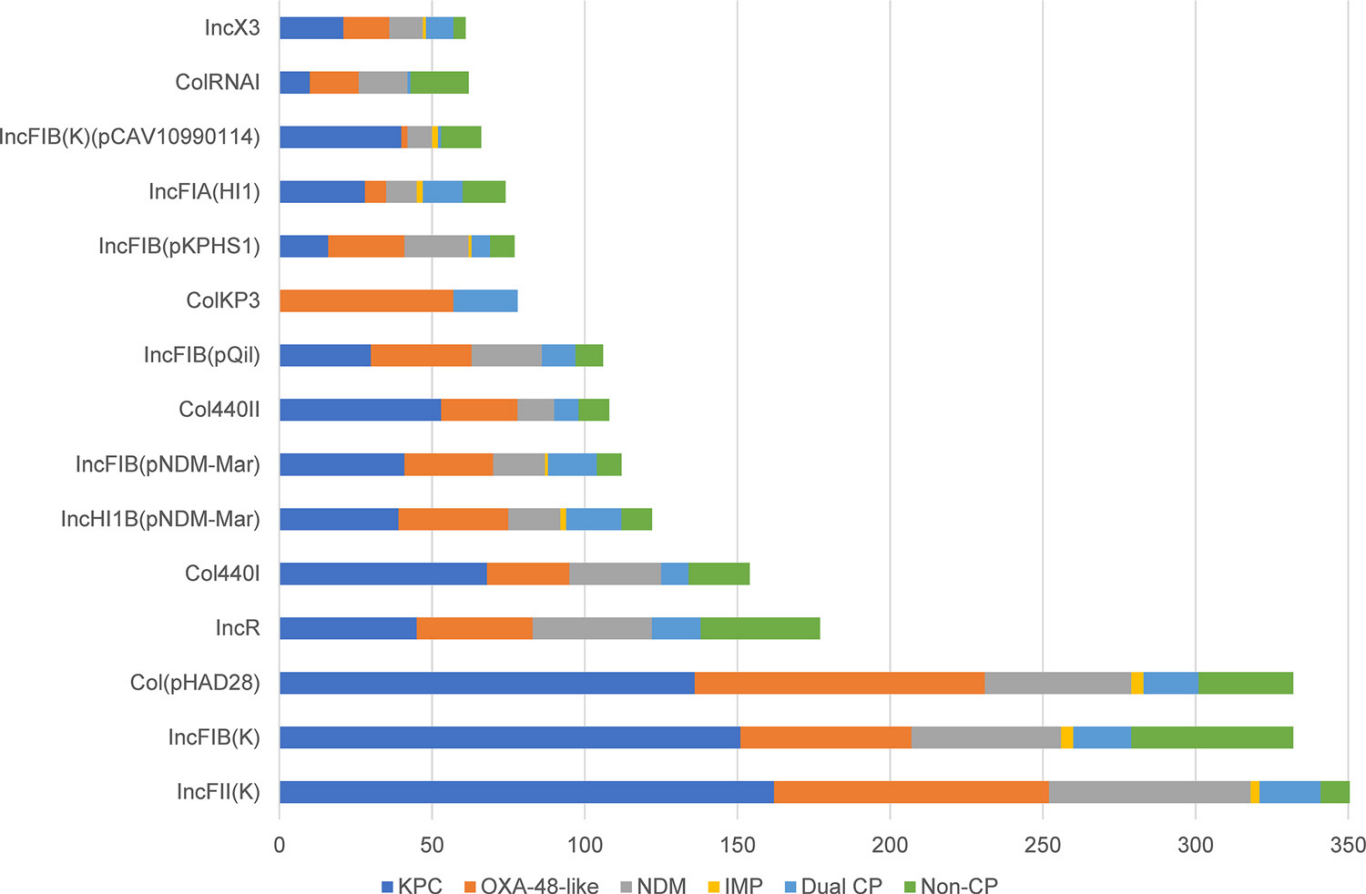
Frequency of plasmid replicon types in carbapenem-resistant K. pneumoniae.

The plasmid analyses identified highly diverse incompatibility (Inc) groups (*n* = 53) within the population surveyed. The frequencies of the various replicon types are shown in Fig. 3. The number of replicons detected in each isolate ranged from 1 to 11 (median: 6), suggesting that some of our isolates might carry multiple plasmids, therefore resulting in the high prevalence of acquired resistance genes from different classes observed in this study.

Unfortunately, due to the limitations of short-read sequencing, we were unable to conduct detailed plasmid investigations. Based on our analyses, we were unable to detect the replicon types for all of the *bla*_KPC_-containing contigs using PlasmidFinder, which may be related to the inability of the current typing scheme to detect all known plasmid types or the contiguous rearrangement/mutations in the regions used for plasmid typing. Here, the majority of the *bla*_KPC_ appeared to be driven by non-Tn*4401* elements; most KPC sequences (234/237, 98.7%) mapped to the non-Tn*4401* prototype sequence pFP10-1 (GenBank: HQ651092.1), concurring with previous observations (17, 28). Additionally, we observed that 216/237 (91.1%) isolates also had sequence reads that mapped to that of a previously identified local *bla*_KPC_ plasmid, pKPC2_sg2 (GenBank: MN542378.1) (23).

Similarly, we were unable to identify the replicon types of the *bla*_NDM_-containing contigs for many samples (89/128, 69.5%). Among the replicons identified, the broad-host range IncN2 replicons were the most frequently detected (31/128, 24.2%). All 31 samples (17 different STs) had reads that mapped to pNDM-ECS01 (GenBank accession: KJ413946.1), which was previously described in NDM-producing E. coli and K. pneumoniae in Singapore (29). Other minority replicons identified included IncC, IncFIB, IncFII, IncHI1B, and IncX3. Only one isolate (ST147) mapped to the pSg1-NDM plasmid (GenBank accession: CP011839.1), which was associated with an interinstitutional outbreak in Singapore (29).

On the other hand, most of the contigs containing *bla*_OXA-181_ or *bla*_OXA-232_ were associated with ColKP3 plasmid replicons (78/118, 66.1%), while a smaller subset carrying *bla*_OXA-181_ were associated with IncC replicons (19/118, 16.1%). The strong association between the ColKP3 plasmid and *bla*_OXA-232_ has been confirmed previously (30). We could not identify the replicons within the remaining five *bla*_OXA-181_ contigs or the replicons within all 43 *bla*_OXA-48_ contigs with PlasmidFinder.

Read-mapping to the pKP3-A reference plasmid (GenBank accession: JN205800), the plasmid bearing *bla*_OXA-181_ isolated from K. pneumoniae and recovered from a patient in Oman (31) revealed its presence in 62/67 (92.5%) of the *bla*_OXA-232_-carrying samples and in 7/51 (13.7%) of the *bla*_OXA-181_-carrying samples, accounting for 42.9% (69/161) of all of the *bla*_OXA-48-like_ samples. pKP3-A was not detected in any of the *bla*_OXA-48_ samples; instead, read-mapping identified the presence of the pOXA-48a IncL/M plasmid (GenBank accession: JN626286), albeit in only 20/43 (46.5%) of our *bla*_OXA-48_ isolates.

### Analyses of the major clones.

**FIG 4:**
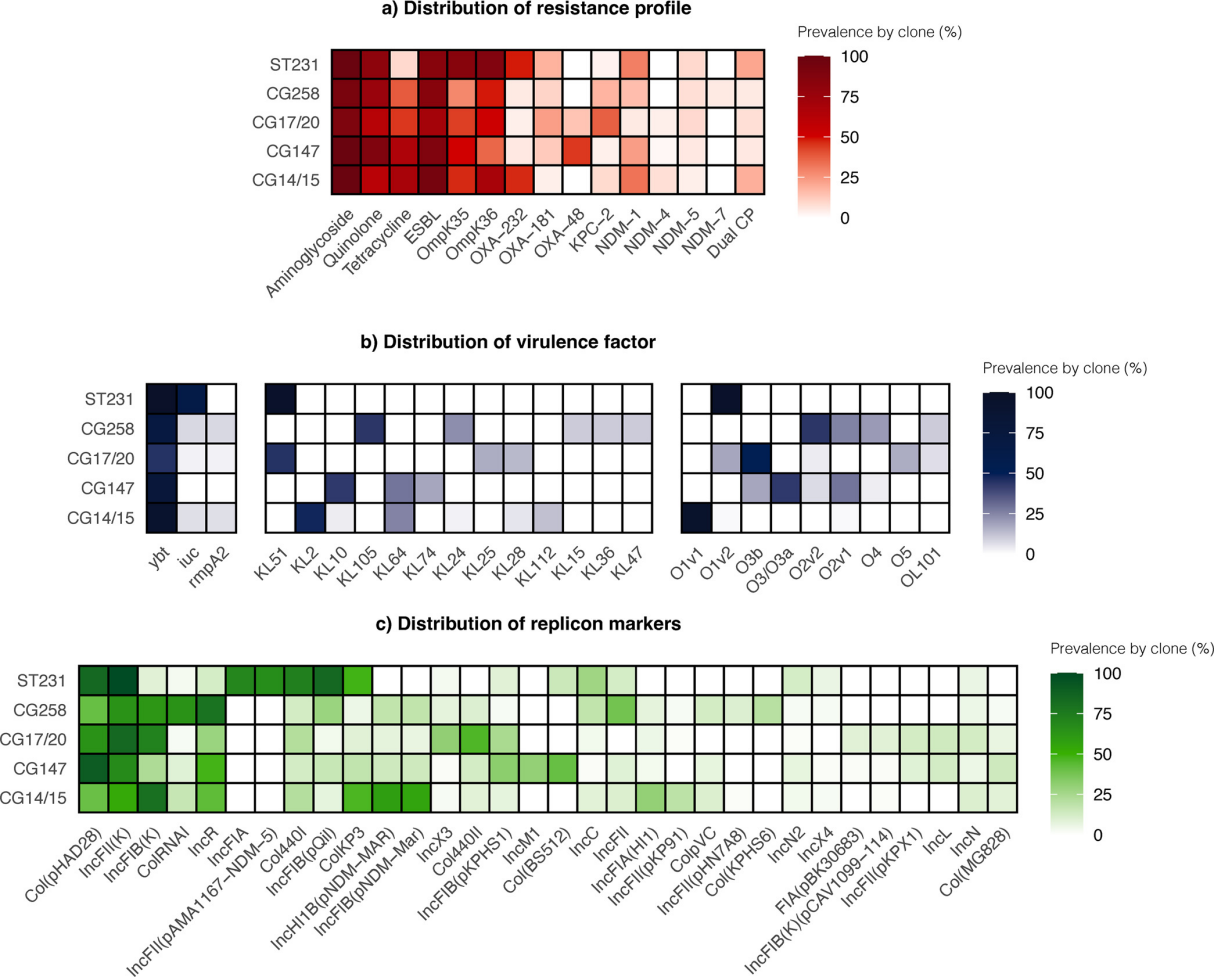
Clonal group heatmaps of carbapenem-resistant K. pneumoniae displaying distributions of (a) key resistance mechanisms. The aminoglycoside, quinolone, and tetracycline groups refer to acquired genes conferring resistance to the antibiotic group. (b) Virulence factors, K locus, and O locus. (c) Plasmid replicons. The values represent the proportions of isolates within each clonal group bearing the individual genetic elements displayed on the horizontal axis. Factors/elements with overall prevalence <10% are not displayed in this figure. Abbreviations: CG, clonal group; CP, carbapenemase-producing; ESBL, extended-spectrum *β*-lactamase; ST, sequence type.

We also sought to explore the major clones in our study. The prevalence of the major classes of AMR determinants, virulence factors, and plasmid replicons within the various CGs is depicted in Fig. 4. Generally, we did not observe any strong overall pattern of association of genomic characteristics with the individual lineages; the various resistance, virulence and plasmid markers were distributed among the major lineages.

**FIG 5:**
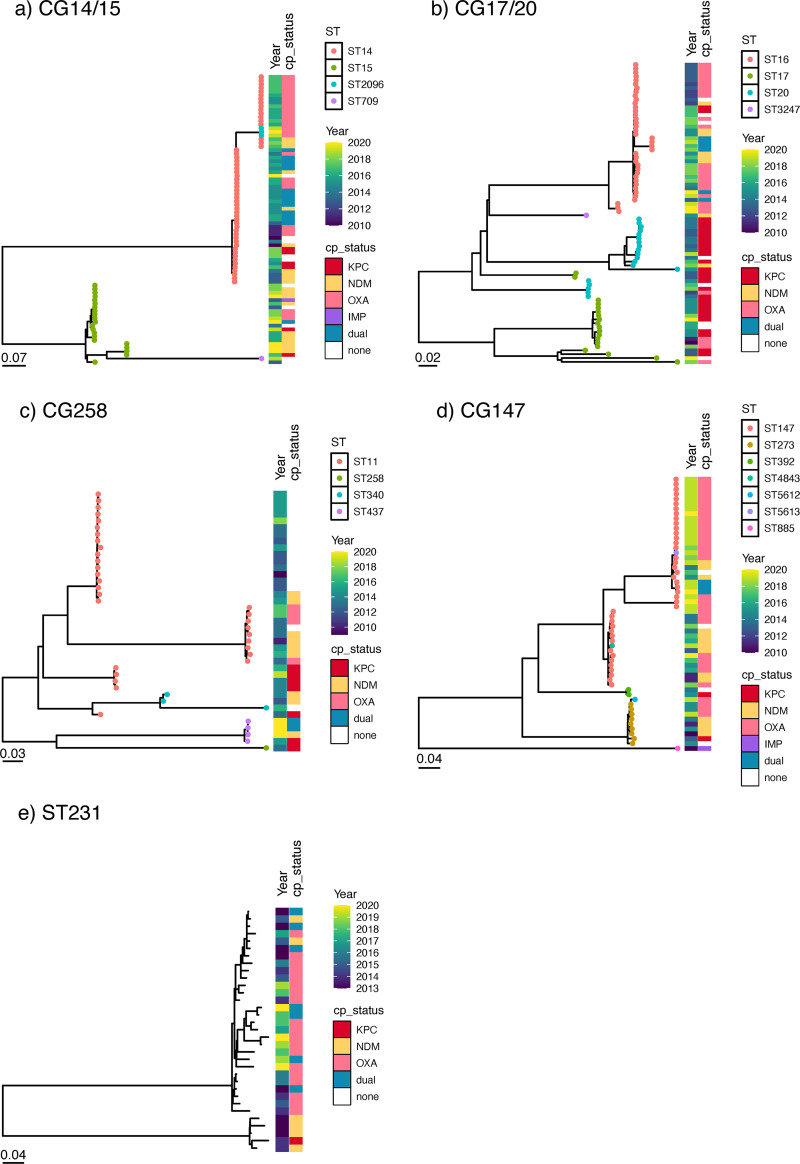
Core SNP phylogenetic trees of major carbapenem-resistant K. pneumoniae lineages. (a) A maximum likelihood tree of CG 14/15 was inferred from the mapping of 79 genomes to reference EC1155. (b) A maximum likelihood tree of CG 17/20 was inferred from the mapping of 78 genomes to reference EC0720. (c) A maximum likelihood tree of CG258 was inferred from the mapping of 37 genomes to reference EC3204. (d) A maximum-likelihood tree of CG147 was inferred from the mapping of 56 genomes to reference EC0325. (e) A maximum-likelihood tree of ST231 was inferred from the mapping of 33 genomes to reference EC2886. Abbreviations: CG, clonal group; CP, carbapenemase producer; SNP, single nucleotide polymorphism; ST, sequence type.

Genomic diversity within each ST is also demonstrated as indicated by the different clades on the individual CG/ST phylogenetic trees (Fig. 5). There were subsets of strains with pairwise SNP differences of ≤25 (Fig. S3A–E) (32). These clusters may represent putative clonal transmission events and should be further confirmed with clinical epidemiological findings.

**FIG 6:**
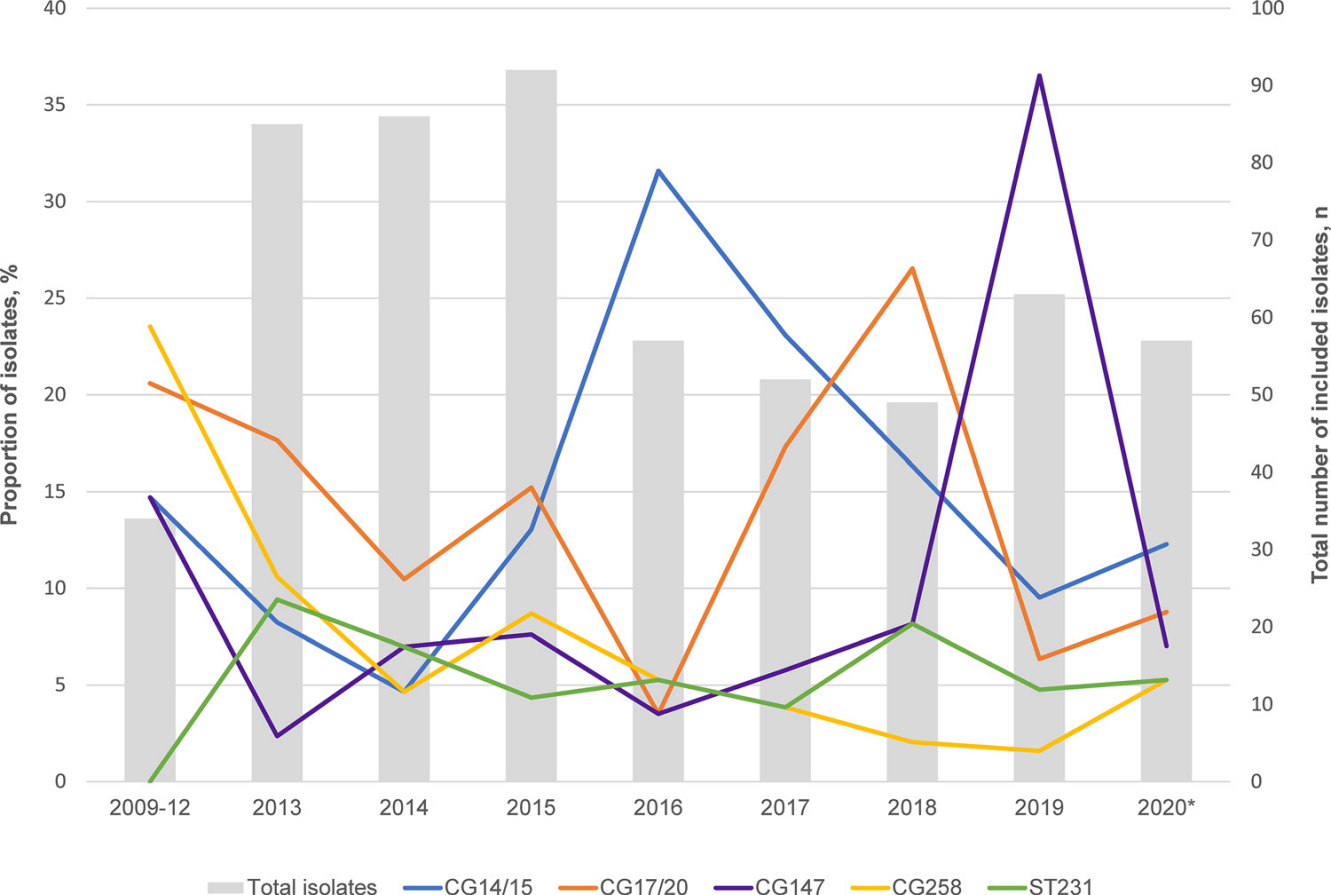
Temporal distribution of the major circulating carbapenem-resistant K. pneumoniae lineages. The primary axis (line graph) denotes the proportion of each CG/ST of the 575 isolates which was included for sequencing. The secondary axis (bar graph) denotes the number of included isolates per year. Isolates from 2009 to 2012 were pooled due to the low number of isolates collected in that time period.

However, it was striking that there was a convergence of resistance and hypervirulence elements in ST231 K. pneumoniae, a lineage more commonly associated with multidrug resistance (Fig. 4). ST231 was first recorded in our collection in 2013, and its prevalence remained stable through the study period (Fig. 6). More than half of the ST231 isolates (21/33, 63.6%) were assigned virulence scores of 4. Additionally, these ST231 isolates exhibited conservation of the K-locus and O-locus (KL51:O1V2), in contrast to the diversity of serotypes observed with the other lineages (Fig. 4B). Furthermore, many of these ST231 isolates carried *bla*_OXA-181_ or *bla*_OXA-232_ on similar ColKP3 plasmid backbones, although the possibility of a clonal expansion was not supported by the phylogenetic analyses, as most pairs had SNP distances of >25 (Mean pairwise SNP distance: 286, range: 6 to 958) (Fig. 5e; Fig. S3E).

**FIG 7:**
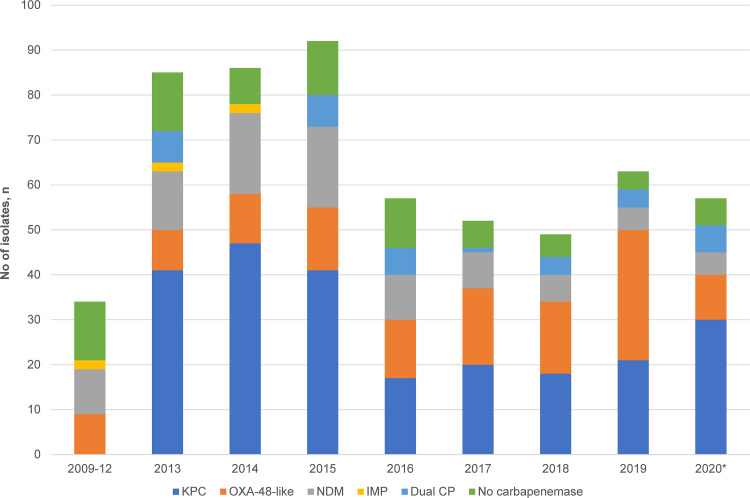
Temporal distribution of carbapenemases in carbapenem-resistant K. pneumoniae. Isolates from 2009 to 2012 were grouped together, as cases were few and collection was sporadic. *Does not include data for the full year.

Another noteworthy finding was the spike in isolates belonging to CG147 in 2019, primarily driven by ST147 (Fig. 6). This may also have contributed to the increasing trend in *bla*_OXA-48-like_ in the recent years, since more than half of ST147 CRKP (30/41, 73.2%) harbored the gene with/without NDM (Fig. 7; Fig. 5D). The phylogenetic analyses suggested the possibility of an undetected transmission cluster, although this requires verification with clinical epidemiological evidence (Mean pairwise SNP difference: 13, range: 9 to 16) (Fig. 5D; Fig. S3D).

## DISCUSSION

In this study, we utilized WGS concurrently with phenotypic surveillance to characterize CRKP isolates collected from a large tertiary public health hospital in Singapore. To our knowledge, this is the largest WGS-based survey of CRKP in Singapore. This study serves to complement and update the growing body of epidemiological findings regarding this pathogen in Singapore.

Circulating CRKP in the institution are highly diverse, composing a polyclonal population structure represented by several well-known international, high-risk clones (ST231, CG147, CG258, CG14/15, and CG17/20) (24). Our findings indicate the limited role of clonal transmission, as we observed only 2,448 of the 330,625 pairs (0.7%) possessing SNP differences below the threshold of 25. It appears that infection prevention measures, such as targeted surveillance, isolation precautions, and strict hand hygiene, were successful in controlling CRKP transmissions between patients residing in the hospital wards, resulting in the stabilization of CRKP incidence rates after 2013. Nevertheless, the acquisition of carbapenem resistance could have also occurred through indirect ward/hospital contact, which was not as easily detected. This hypothesis is supported by the findings from another local paper, which identified an independent association of indirect contact with putative clonal transmissions (33). Furthermore, the larger contribution of plasmid transmission toward carbapenem resistance dissemination may be related to non-human reservoirs, such as the hospital environment, which has been identified in previous studies as an important source of MDR organisms. Future control efforts should be directed to environmental surveillance that includes high-risk areas, such as sinks and toilets, to identify hidden CRKP reservoirs (34, 35).

In a previous study of acquired carbapenemases in the institution from 1996 to 2012, ST14 K. pneumoniae was the most common strain that contributed to carbapenem resistance in the institution (36). *bla*_OXA-181_-producing ST14 strains have also been detected in other public hospitals in Singapore (37). In our study, CG14 (comprising largely of ST14 and ST15) was observed to be one of the predominant carbapenem-resistant clonal groups over the study period. CG14 represents the most common third-generation, cephalosporin-resistant clones in various regions, particularly on the Asian continent (19, 38). This might explain its high prevalence (13.7%) in this study, considering the endemicity of ESBL-producing Enterobacterales in our region. Carbapenem resistance has emerged in this clonal complex and has been described in isolates producing the OXA-48-type with or without NDM carbapenemases in Tanzania, South Korea, the United Arab Emirates, and Turkey (19, 39–42). While some of the earliest cases of ST14 carbapenemase producers (observed in 2001) were reported to have been isolated from patients with travel history to Bangladesh, where OXA-181-producing Enterobacterales are endemic (43), it is likely that circulating CG14 strains that have established a successful foothold in the local setting have also acquired different carbapenemases (including KPC, IMP, OXA-48, and and NDM) over multiple independent events, as exemplified by the large pairwise SNP differences observed with the ST14 isolates.

Likewise, the CG258 subclone ST11 is historically one of the most common carbapenemase-carrying clones in the local population (36). ST11 K. pneumoniae is the dominant KPC clone in China, and it is the single-locus variant of ST258, the widely circulating KPC-2/3 clone in the United States and parts of Europe (Italy and Greece) (44). ST11 constituted the majority of the CG258 strains in our study; there was only one occurrence of the ST258 strain which was a KPC-2 carrier, while the ST512 strain was absent. A 2011 study involving four KPC-producing isolates from various Singapore hospitals identified that all strains belonged to ST11, suggesting that the early dissemination of KPC locally may be attributed to ST11 (28).

However, we found that KPC enzymes were later disseminated via diverse clones over the study period. KPC carriage was not independently associated with any of the prevalent clones in this study, and it often occurred in non-CG258 strains. CG258 accounted only for 7 (2.9%) of the 235 KPC-carrying isolates. Instead, the CG258 strains were either non-carbapenemase producing or carriers of NDM/OXA-48 enzymes. This is in concordance with the findings by the CaPES group, which attributed KPC-2 prevalence in Singapore public health hospitals primarily to non-CG258 isolates (17). Furthermore, the findings here point toward the likelihood of local KPC dissemination being mediated by a few successful plasmids transmitted across multiple different lineages, as evidenced by majority of the KPC-containing contigs mapping to a local *bla*_KPC_ plasmid. This observation is also supported by various other local studies and may explain the predominance and endemicity of KPC enzymes locally (17, 23).

Our study also highlights the emergence of several notable high-risk lineages. ST231 is an emerging CRKP epidemic clone which has been reported in several countries, including India, Thailand, Brunei, France, and Switzerland (45, 46). In India, there were several reports describing this lineage as one of the predominant STs in the region (47, 48). Our isolates share common genomic characteristics with these previously described ST231 strains; they were MDR and mostly carried *bla*_OXA-181/232_, *bla*_CTX-M_, 16S RMTases (*arm*A/*rmt*B/*rmt*F), porin mutations, and the virulence genes, *ybt* and *iuc*. Cocarriage of *bla*_NDM_ was also frequently observed, conferring high levels of resistance. The convergence of virulence and resistance elements (including 16S RMTases with the ability to hydrolyze new aminoglycosides, such as plazomicin) in this emerging CRKP high-risk clone requires high vigilance, as this may represent an important reservoir of genetic elements which may be horizontally transferred to other lineages.

We also observed ST147, which is an emerging epidemic clone capable of disseminating various resistance elements. This clone likely emerged in Europe in the early 1990s, where it acquired *gyr*A and *par*C mutations following the introduction of ciprofloxacin, and it is associated with *bla*_CTX-M-15_. In the late 2000s, the clone came to be associated with the various carbapenemases, according to the geographical regions in which specific carbapenemases prevailed (e.g., NDM and OXA-181 in India and KPC in Greece), allowing the clones to establish themselves as endemic pathogens in these locales (49). The ST147 strains in our study were most frequently associated with OXA-48-like enzymes. Additionally, the genetic relatedness of the strains suggests local clonal expansion, which may explain the increasing trend of ST147 and the corresponding increasing prevalence of OXA-48-like enzymes from 2018 to 2020. Interestingly, this clone has been implicated in a local interinstitutional transmission of *bla*_NDM_ on a novel plasmid, pSG1-NDM, previously, although we only detected the plasmid in one sample within our collection (29).

There is evidence of recent OXA-48-like outbreaks that involved multiple Enterobacterales within the hospital (50). While the clonal transmission of ST147 may have contributed to the displacement of the other carbapenemases by OXA-48-like enzymes, horizontal transmission of OXA-48-plasmids likely played an important role in the increasing prevalence of OXA-48 in the institution, as it can even spread to/via other species. Previous studies have shown that *bla*_OXA-48_ dissemination in certain regions, such as the Mediterranean and Western Europe, was a consequence of the successful spread of single plasmids. Different clones from different geographical areas, and even different Enterobacterales species, carried *bla*_OXA-48_ on plasmids that shared similar genetic features/plasmid backbones. These were typically self-conjugative plasmids of 60 to 70 kb in size (51–54). Similar observations exist for the *bla*_OXA-232_ plasmids. There were several reports documenting the carriage of *bla*_OXA-232_ on ~6kb plasmids with ColE/KP3-like backbones in isolates from India, the United States, and China, and these plasmids often showed high similarity to the *bla*_OXA181_-bearing pKP3-A plasmid from Oman, which was also found in high frequency in our study (31, 55, 56).

This study is not without limitations. Sample collection and sequencing were not exhaustive, due to resource constraints. As surveillance was not largely mandated or standardized, we were reliant on the sampling criteria and procedures of the institution. Surveillance isolates were tested for carbapenamase production using GeneXpert Xpert Carba-R, and they were not consistently cultured out after 2015, resulting in the uneven distribution of surveillance isolates throughout the study period. The lack of systematic sampling could potentially have introduced unintentional sampling bias. Moreover, the exclusion of antibiotic-susceptible strains and the lack of such existing local info impeded our analysis of the evolution of the AMR strains. As the study was conducted in a single center, the results here may not be generalizable to the local population at large. In addition, the lack of clinical epidemiological information prohibited us from establishing conclusive evidence regarding transmission clusters, as epidemiologically unrelated samples could also display little genetic variability at the SNP level. Lastly, we relied on short-read sequencing, which is limited in its resolution, in the investigation of the mobile genetic elements.

### Conclusions.

Through this study, we have demonstrated the utility of WGS in improving the understanding of AMR transmission. We established that the local population structure of CRKP encompasses well-known epidemic, MDR clones amid a diverse pool of nonepidemic lineages. The multiple phylogenetic clusters of the major circulating clones here primarily demonstrated multiple independent acquisition events, as opposed to clonal expansion/transmission. However, subsets of strains found to be clustering together may point toward outbreaks/reservoirs (e.g., ST147) which otherwise would not have been resolved via conventional typing methods. Furthermore, the plasmid analyses illustrated a divergence in the transmission dynamics of the three carbapenemases. Further exhaustive analyses could potentially uncover the local evolutionary perspectives of these carbapenemases.

The comprehensive resistance, virulence, and plasmid gene content profiling provided critical information for use in AMR monitoring and highlighted future surveillance priorities. While problematic multidrug resistance elements, such as *mcr* and *tet*(X), remained rare, the emergence of ST231 K. pneumoniae bearing multidrug resistance genes (including those that confer resistance to novel agents) and virulence elements concurrently warrants closer attention. The seemingly increasing *bla*_OXA-48-like_ trend is also concerning and deserves ongoing surveillance. The establishment of this detailed hospital-specific repository will not only enable more precise and rapid analyses of future isolates to allow for the timelier identification of areas of concerns and corresponding interventions, but also serves to inform future treatment guidelines and research priorities.

Lastly, the findings here reinforce the necessity of unique infection control and prevention strategies that take into consideration the genomic diversity of local circulating strains. We should be mindful that strategies developed or employed in geographical areas with a limited number of circulating clones may not necessarily be adopted here effectively.

## MATERIALS AND METHODS

### Study setting.

This study was conducted at the Singapore General Hospital, the largest public acute care tertiary hospital in Singapore. The hospital has approximately 1,800 beds and accounts for approximately 25% of the total acute hospital beds in the public sector and 20% of the acute beds nationwide. A wide range of medical and surgical specialties are offered by the hospital, and it is the national and regional referral center for services such as plastic surgery, burns, renal medicine, nuclear medicine, pathology, and hematology.

Specific interventions targeted at carbapenemase-producing Enterobacterales control included the active surveillance of carbapenemase carriage in high-risk patients (hospitalization in the preceding year; admission to intensive care, renal, hematology, or oncology units; and a duration of hospital stay of ≥2 weeks) and contact precautions for colonized/infected patients (isolation in single/cohort rooms). The targeted surveillance of carbapenemase-producing organisms only commenced in the hospital after 2013 (50, 57). The incidence rates of CRKP in the hospital over the study period are displayed in Fig. S1.

### Bacterial isolates.

A total of 575 CRKP isolates from 547 adult inpatients collected between 2009 and 2020 were available for WGS and were included in the analyses. Only one isolate was included per patient per year, unless the isolates displayed different genotypic characteristics (e.g., different carbapenemases). The sequenced isolates represented approximately 75% and 14% of the clinical and surveillance CRKP isolates collected over the study period, respectively (Fig. S2). Carbapenem resistance was defined as nonsusceptibility to at least one carbapenem (ertapenem, doripenem, meropenem, or imipenem). These isolates were collected at the institution’s Microbiology Laboratory and tested at the Pharmacy Research Laboratory as part of an informal surveillance study of carbapenem nonsusceptible Gram-negative organisms. Isolates received at the Pharmacy Research Laboratory for antibiotic combination testing were also included. The collection included both routine surveillance (*n* = 132, 23.0%) and clinical isolates (*n* = 443, 77.0%) from various sources (urinary, blood, gastrointestinal/abdominal, wound, respiratory, and bone). Generally, invasive isolates from sterile sites were prioritized for WGS.

All isolates were identified using Vitek GNI+ cards with the Vitek 2 instrument (bioMérieux, Hazelwood, MO, USA) and/or a matrix-assisted laser desorption/ionization time-of-flight mass spectrometry (MALDI-TOF MS) system (Bruker Daltonik, Germany) as part of the routine workflow of the institution’s microbiology laboratory. Only isolates which were eventually identified *in silico* as K. pneumoniae *sensu lato* were included in the analyses.

### Antibiotic susceptibilities.

MICs were obtained using customized 96-well broth microdilution panels (TREK Diagnostics, East Grinstead, UK) in accordance with the manufacturer’s recommendations and were interpreted according to the Clinical & Laboratory Standards Institute (CLSI) breakpoints (58), except for tigecycline, which was interpreted according to the US FDA criteria. Fosfomycin MICs were obtained using gradient MIC test strips (bioMérieux, Marcy l’Etoile, France). Escherichia coli ATCC 25922 was used as the quality control strain.

### DNA preparation and whole-genome sequencing.

Overnight bacterial cultures in cation-adjusted Muller-Hinton were prepared and used for genomic DNA extraction using the DNeasy Blood and Tissue Kit (Qiagen GmbH, Hilden, Germany) according to the manufacturer’s instructions. The genomic DNA was then sent for paired-end WGS using MiSeq/HiSeq systems (Illumina Inc., CA, USA), with a resultant sequencing depth of at least 50-fold. Additionally, some of the raw sequences were retrieved from the multicenter study (Carbapenemase-Producing Enterobacteriaceae Study [CaPES]), which involved major public health hospitals in Singapore (BioProject PRJNA342893) (6). Raw sequences were assessed for quality using FastQC (v0.11.3, Babraham Institute), followed by the removal of adaptors and poor-quality bases and sequences using Trimmomatic (59, 60). The trimmed sequences were then assembled *de novo* using the SPAdes software (61).

### Antimicrobial resistance profiling, serotyping, and virulence gene characterization.

Relevant resistance genes were identified using the NCBI-AMRFinderPlus database (v3.10.16) and the Kleborate tool (v.2.0.4) (https://github.com/katholt/Kleborate) (62). The Kleborate tool was also used to identify the ICEKp-associated and plasmid-associated virulence loci (i.e., genes encoding yersiniabactin [*ybt*], aerobactin [*iuc*], salmochelin [*iro*], the colibactin toxin [*clb*], and the mucoid phenotype regulators [*rmpA*, *rmpA2*]). Kleborate assigns a virulence score to each of the samples. Virulence scores are ranked from 0 to 5 based on the presence of *ybt*, *clb*, and *iuc* as follows: 0 = none present, 1 = *ybt* only, 2 = *clb* with/without *ybt*, 3 = *iuc* only, 4 = *iuc* and *ybt*, and 5 = *ybt*, *clb*, and *iuc*. “Convergent” K. pneumoniae are defined as strains which are both hypervirulent (carrying the *iuc* aerobactin locus) and MDR (carrying ESBL or carbapenemase genes) (39).

The Kaptive tool was also used to perform capsular typing, in which capsule polysaccharide (K) and lipopolysaccharide (O) loci were determined (63). Plasmid replicons were identified with PlasmidFinder (64).

### *In silico* multilocus sequence typing and phylogenetic analyses.

STs were identified by BLAST, using the scheme in the Pasteur database (https://bigsdb.pasteur.fr/klebsiella/). Core genome alignment of the assembled K. pneumoniae draft genomes was performed using Parsnp from the Harvest suite of phylogenetic tools (65). The alignment was run with EC0295, which was randomly chosen by Parsnp as the reference genome for the main tree. For the individual CG/ST trees, the reference genomes were EC1155, EC0720, EC3204, EC0325, and EC2886 for the CG14/15, CG17/20, CG147, CG258, and ST231 phylotrees, respectively. The resultant phylogenetic trees were visualized using the R ggtree package (66). The transmission inference threshold used to identify genomically closely related isolates was a SNP difference of ≤25 (32).

### Statistical methods.

Comparisons involving dichotomous variables were tested using a Chi-square test or Fisher’s exact test, as appropriate. No adjustment was made for multiple comparisons, due to the exploratory nature of this study. All statistical analyses were conducted with IBM SPSS Statistics for Windows, Version 26.0 (IBM Corp., Armonk, NY). A two-tailed value of *P* < 0.05 was considered to be indicative of statistical significance.

### Ethics statement.

This study is exempt from review by the Singhealth Centralised Institutional Review Board, as it is a retrospective study involving archival bacterial isolates, which does not fall under the Human Biomedical Research Act. No identifiable data were collected.

### Data availability.

The whole-genome sequences of CRKP used in this study are available in the NCBI Sequence Read Archive (SRA) under BioProject accession number PRJNA577535 (https://www.ncbi.nlm.nih.gov/bioproject/).
