# Supplementary material for: Genomic Surveillance of Carbapenem-Resistant Klebsiella pneumoniae from a Major Public Health Hospital in Singapore
Source: Microbiol Spectr. 2022 Sep 6;10(5):e00957-22. doi: 10.1128/spectrum.00957-22 (PMC9602435; doi:10.1128/spectrum.00957-22)
Supplement: Supplemental file 1 — Fig. S1 to S3. Download spectrum.00957-22-s0001.pdf, PDF file, 3.2 MB [file spectrum.00957-22-s0001.pdf]

Supplementary figures

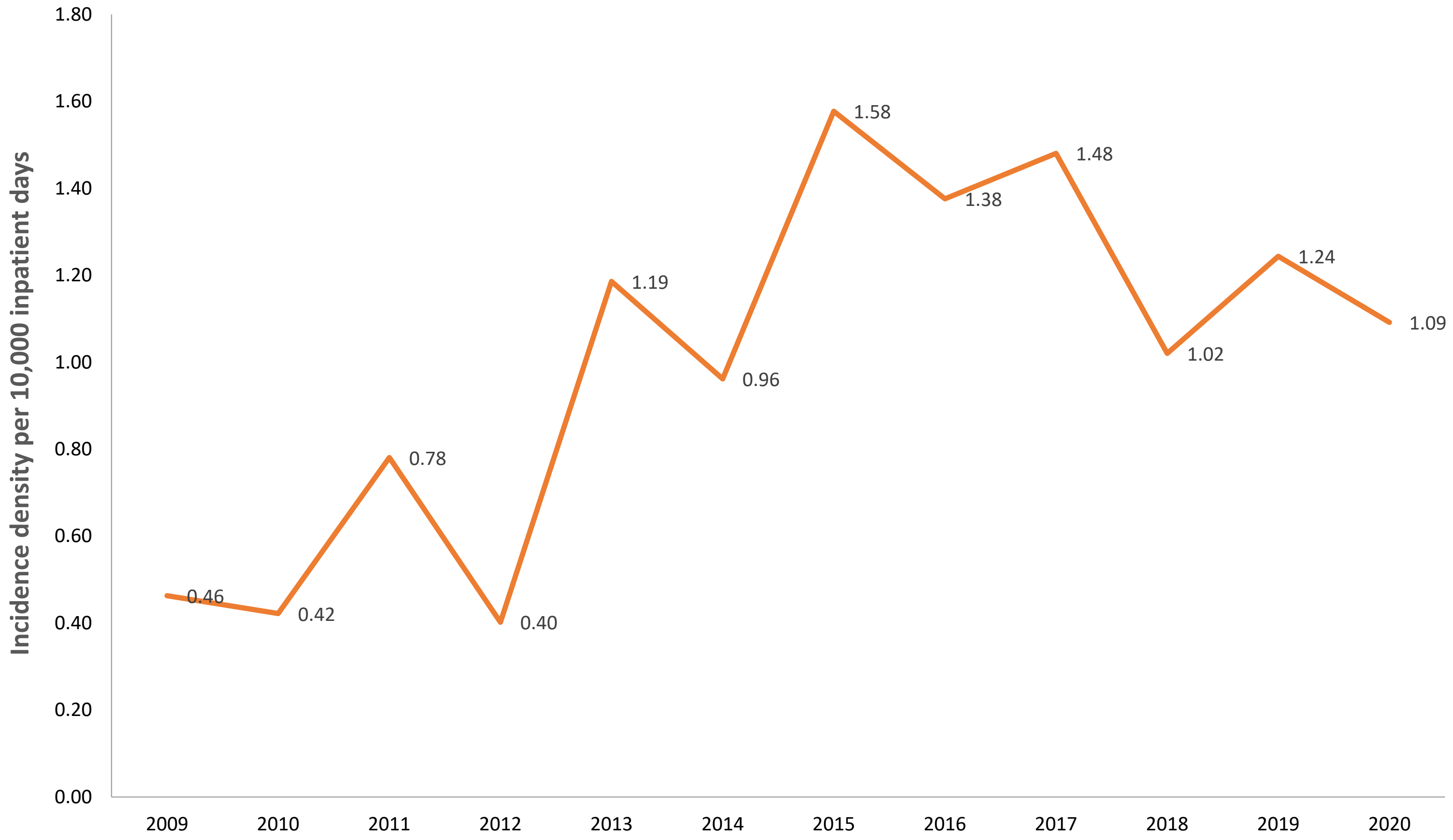

Figure 1. Incidence density trends of clinical carbapenem-resistant *K. pneumoniae* from 2009 – 2020

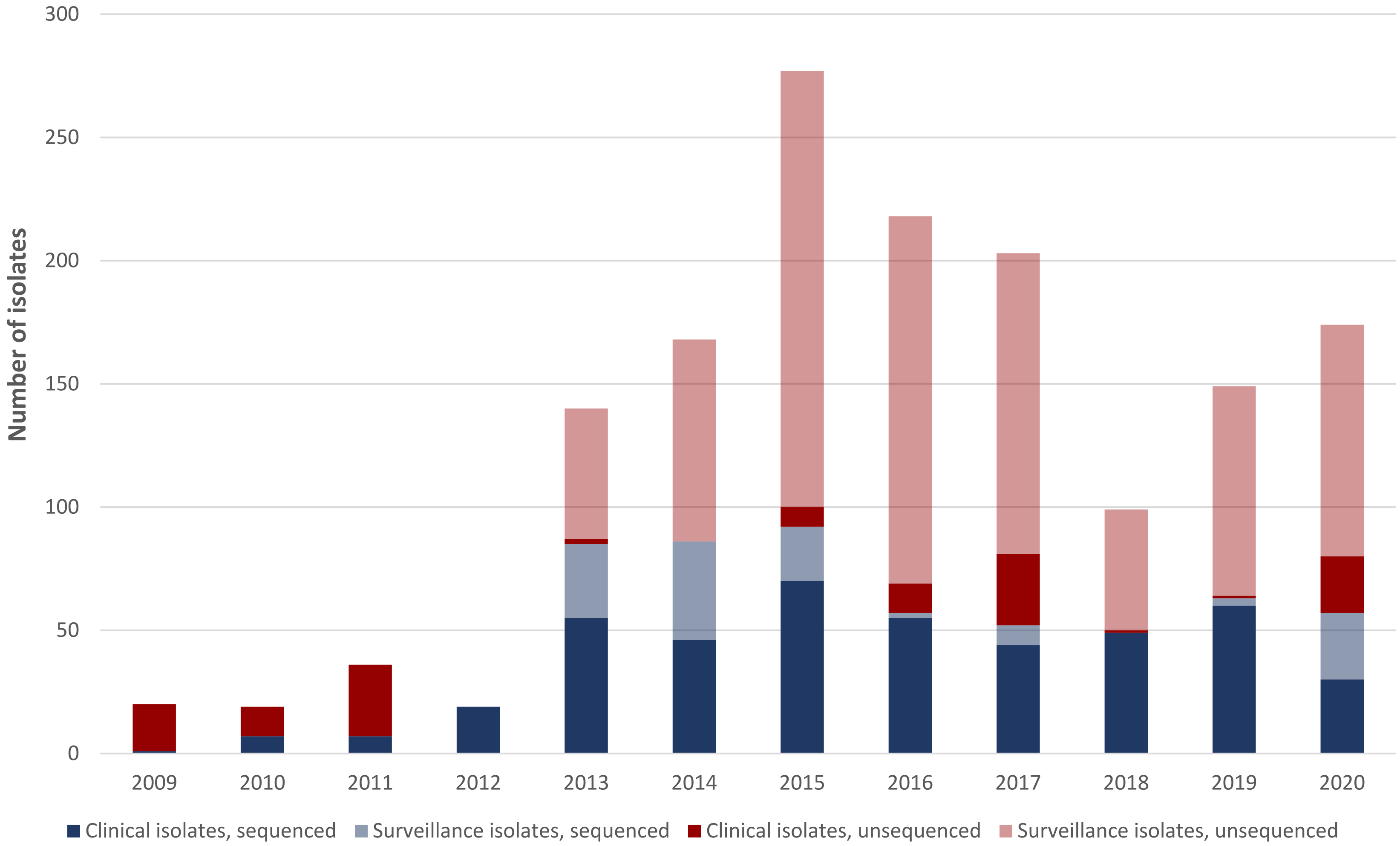

Figure 2. Number of carbapenem-resistant isolates collected and sequenced from 2009 – 2020

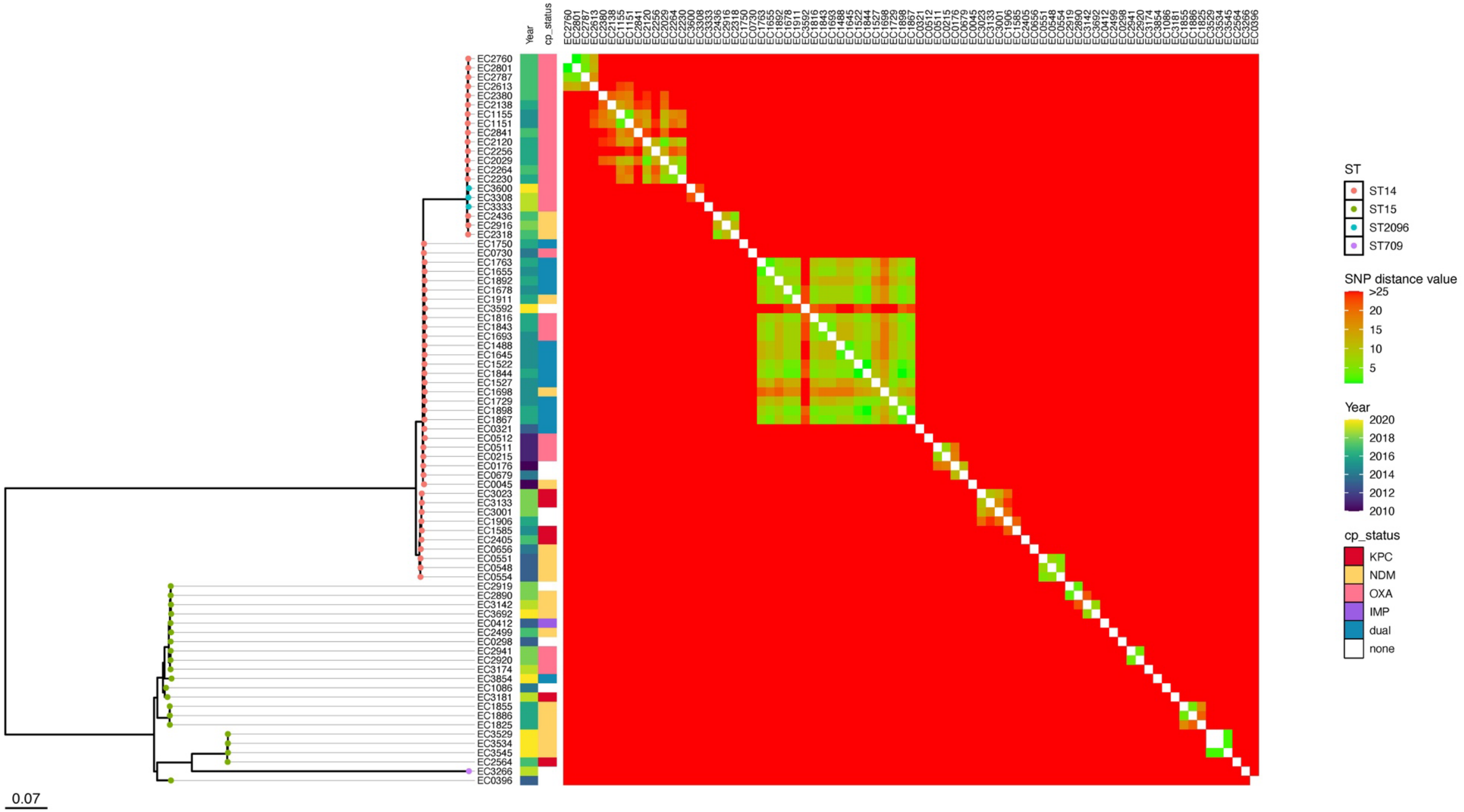

Figure 3a Core SNP phylogenetic tree of CG14/15 genomes with pairwise SNP distance matrix. The maximum-likelihood tree of CG14/15 was inferred from mapping 79 genomes to reference EC1155. The scale bar indicates the number of substitutions per site. CG, clonal group; SNP, single nucleotide polymorphism; ST, sequence type.

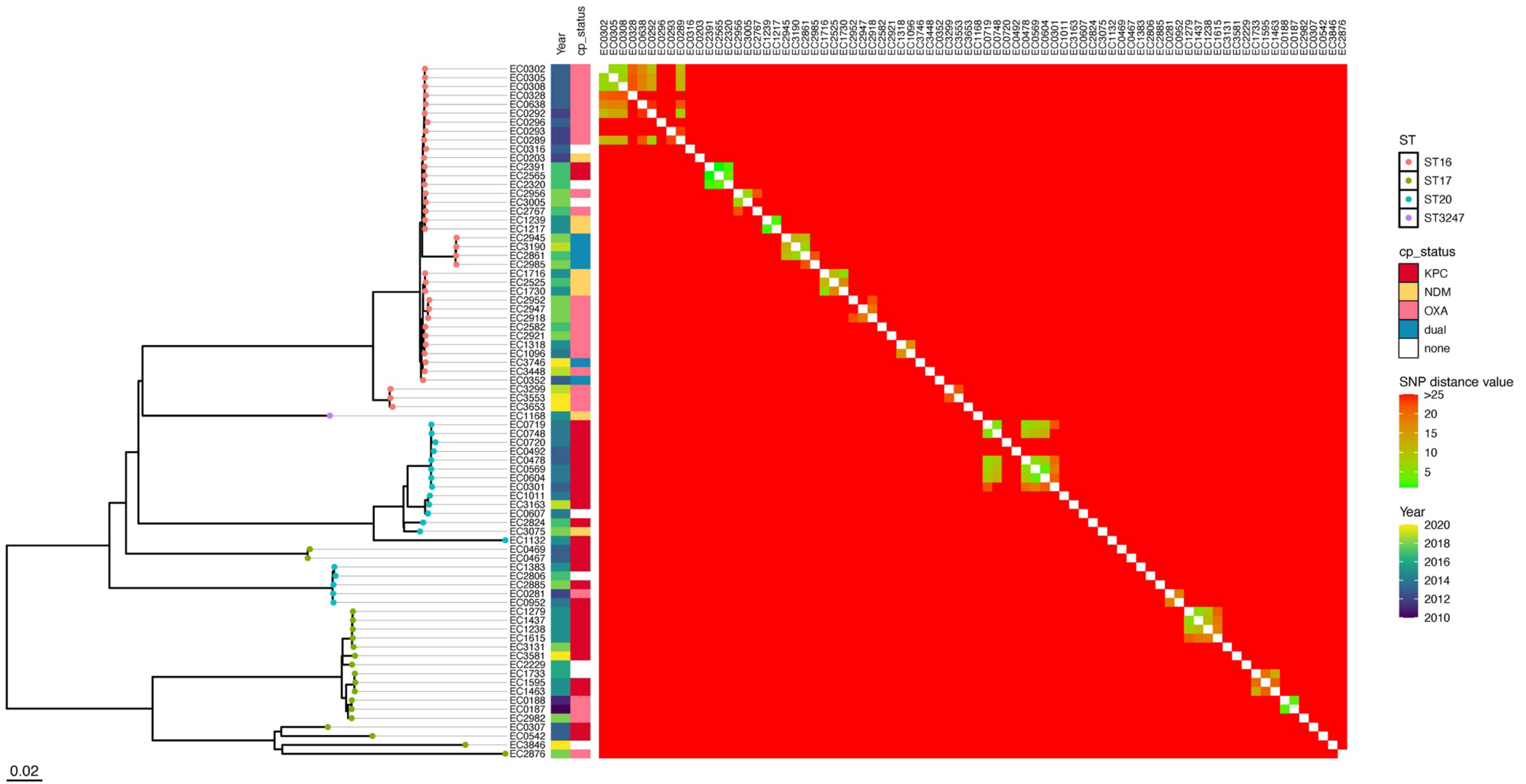

Figure 3b. Core SNP phylogenetic tree of CG17/20 genomes with pairwise SNP distance matrix. The maximum-likelihood tree of CG17/20 was inferred from mapping 78 genomes to reference EC0720. The scale bar indicates the number of substitutions per site. CG, clonal group; SNP, single nucleotide polymorphism; ST, sequence type.

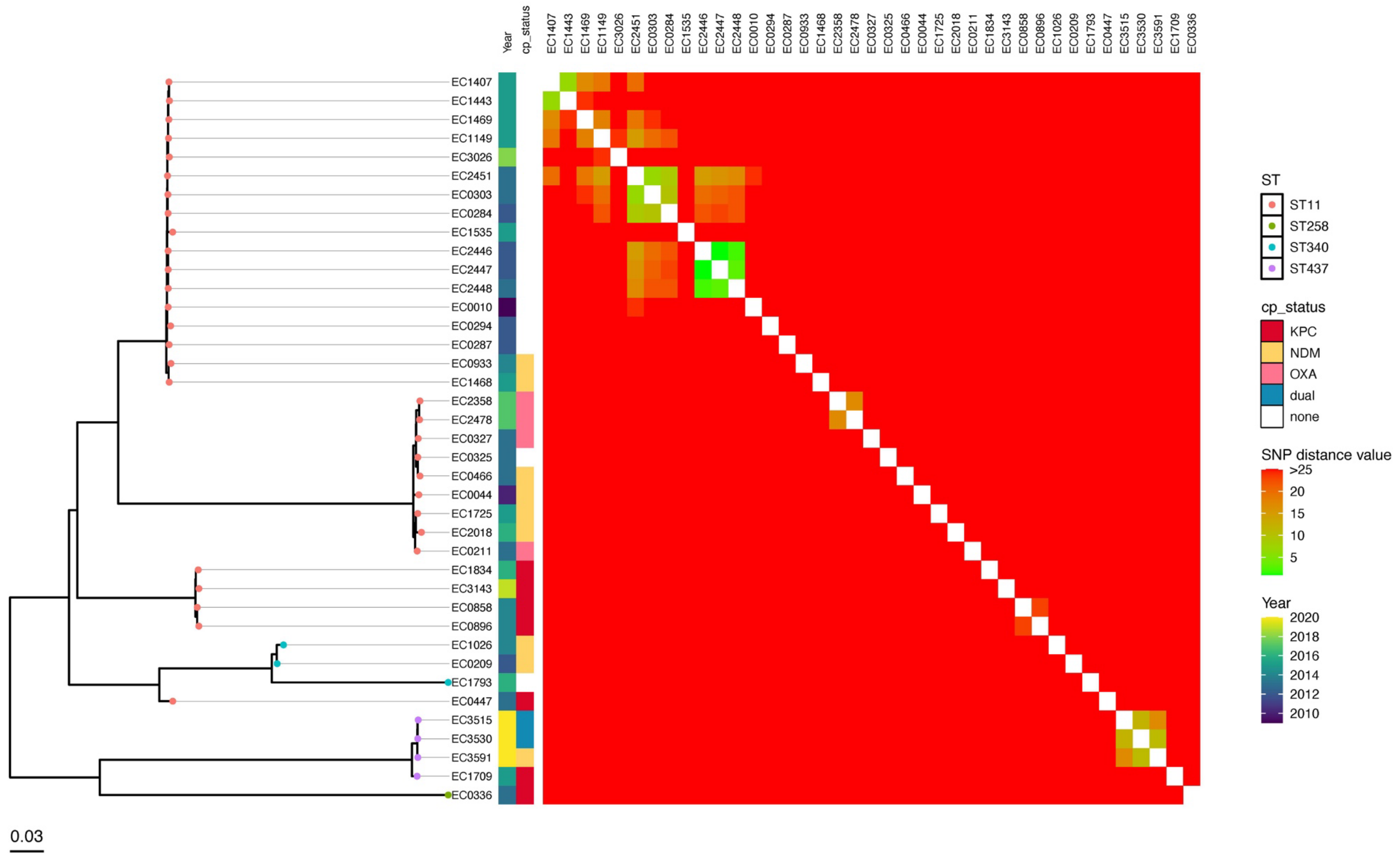

Figure 3c. Core SNP phylogenetic tree of CG258 genomes with pairwise SNP distance matrix. The maximum-likelihood tree of CG258 was inferred from mapping 39 genomes to reference EC3204. The scale bar indicates the number of substitutions per site. CG, clonal group; SNP, single nucleotide polymorphism; ST, sequence type.

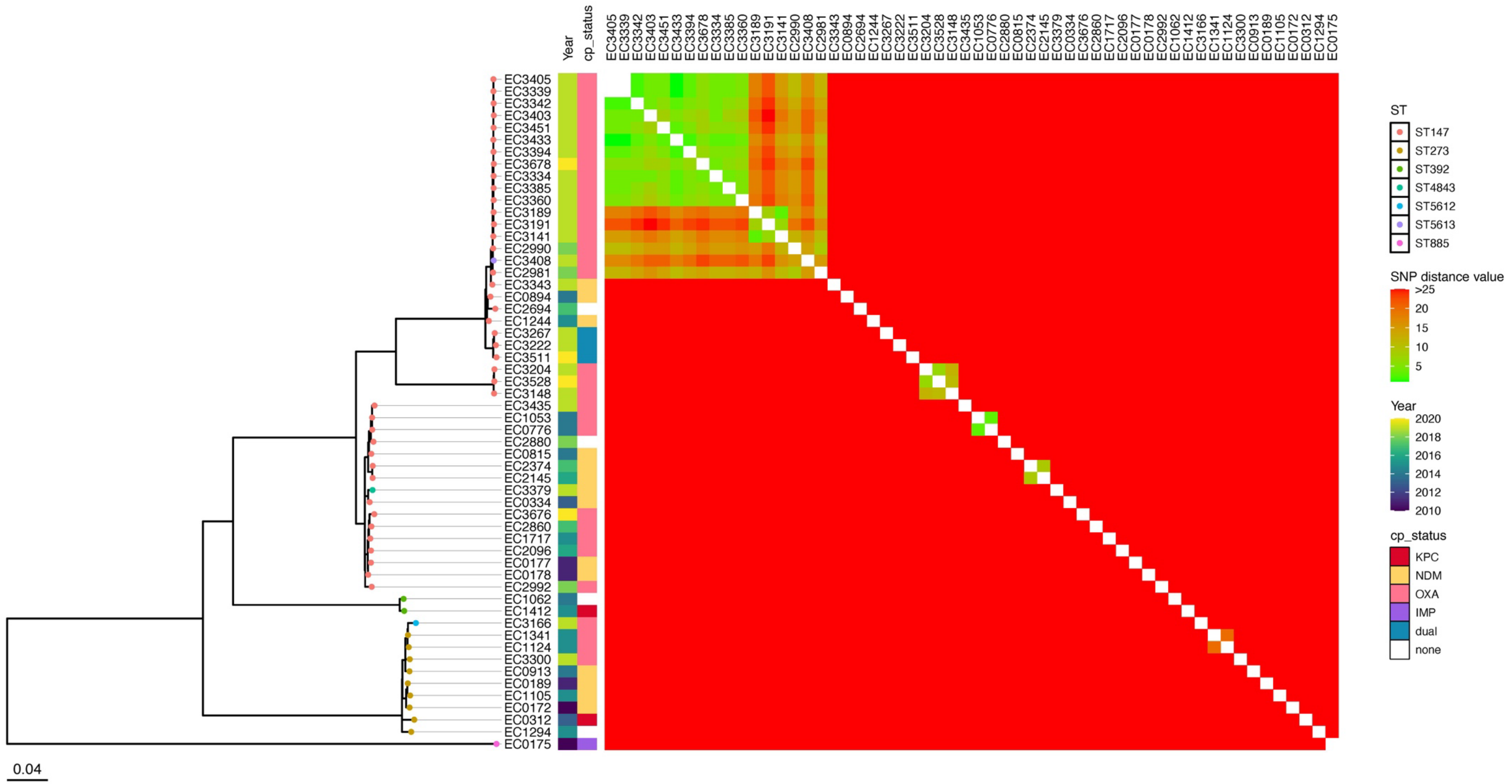

Figure 3d. Core SNP phylogenetic tree of CG147 genomes with pairwise SNP distance matrix. The maximum-likelihood tree of CG147 was inferred from mapping 56 genomes to reference EC0325. The scale bar indicates the number of substitutions per site. CG, clonal group; SNP, single nucleotide polymorphism; ST, sequence type.

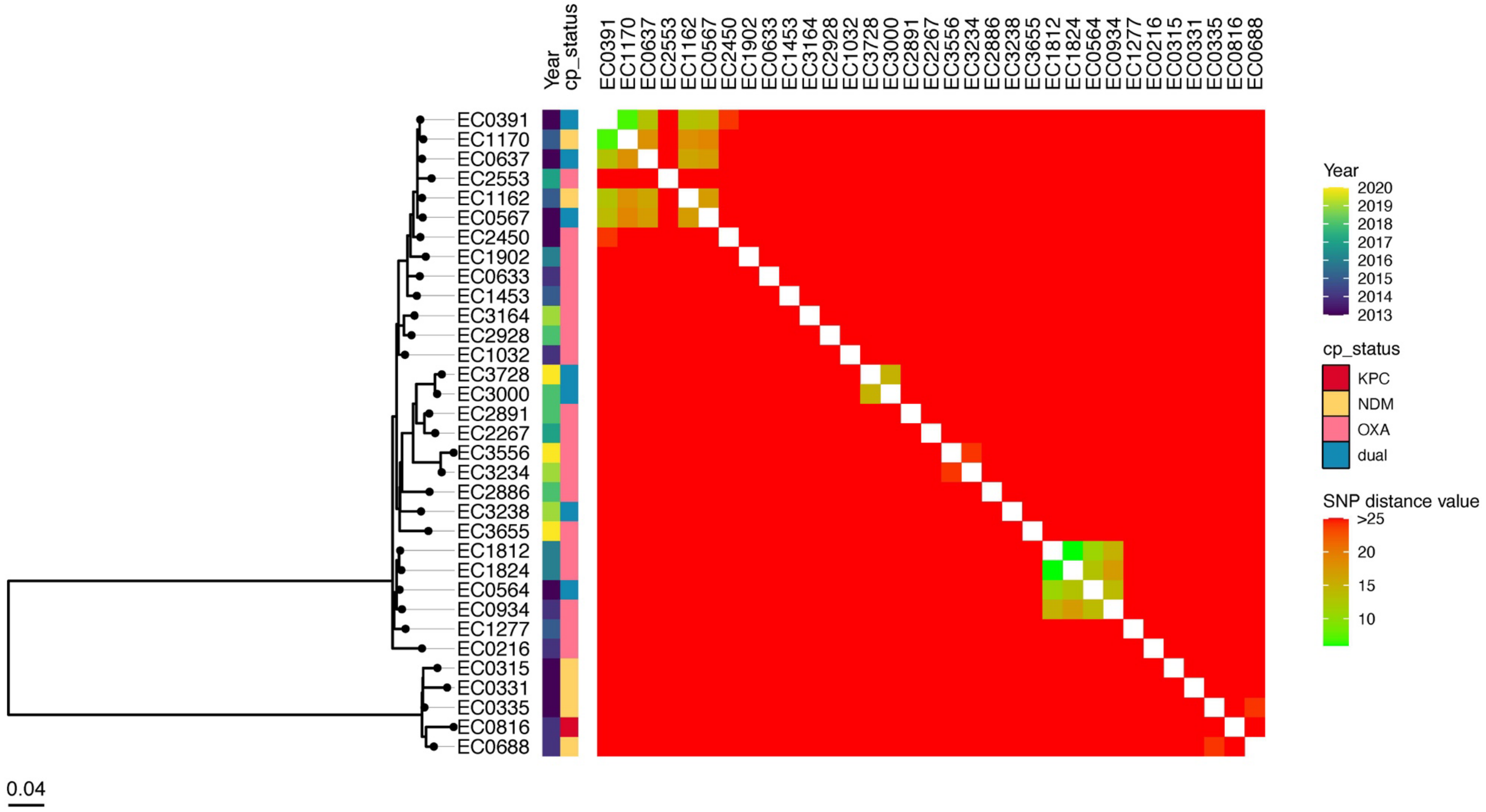

Figure 3e. Core SNP phylogenetic tree of ST231 genomes with pairwise SNP distance matrix. The maximum-likelihood tree of ST231 was inferred from mapping 33 genomes to reference EC2886. The scale bar indicates the number of substitutions per site. CG, clonal group; SNP, single nucleotide polymorphism; ST, sequence type.
